# The Kidd-Quain Consultation

**Published:** 1881-09

**Authors:** 


The Kidd-Quain Consultation.—We are happy to find that the views we expressed upon this matter soon after its occurrence, have been abundantly sustained by the larger number of intelligent members of the profession in England, and in our own country. Indeed, we felt sure such would be the case when the editorial which appeared in our May issue was penned, so soon as the excitement which was fanned into a furious flame, either by accident or design, should have time to die out, and the profession allowed to view the whole transaction in its proper light. That Dr. Quain, instead of being denounced and jeered at for meeting Dr. Kidd in consultation, would be commended and applauded for his humanity and his manliness in stepping to the front in an attempt to rescue the foremost statesman and political leader in England from the grave! Right glad we are to learn that Her Majesty, the Queen, has decided to decorate Dr Quain with high and distinguished honors for his heroic and manly conduct in joining Dr. Kidd in a united effort for the relief of Lord Beaconsfield. We hope that an appeal from humanity will always be held superior to a code of ethics, as manly conduct, based upon correct and proper principles, will be sure of its reward sooner or later. In Dr. Quain's case, the public reward for his humanity has followed very soon after his manly action. Dr. Kidd is in no sense a homœopathist. H. L. B.

